# Intraradicular Splinting with Endodontic Instrument of Horizontal Root Fracture

**DOI:** 10.1155/2015/505370

**Published:** 2015-01-12

**Authors:** Ersan Çiçek, Neslihan Yılmaz, Mustafa Murat Koçak

**Affiliations:** Department of Endodontics, Faculty of Dentistry, Bulent Ecevit University, Kozlu, 67600 Zonguldak, Turkey

## Abstract

*Introduction*. Root fractures, defined as fractures involving dentine, cementum, and pulpal and supportive tissues, constitute only 0.5–7% of all dental injuries. Horizontal root fractures are commonly observed in the maxillary anterior region and 75% of these fractures occur in the maxillary central incisors. *Methods*. A 14-year-old female patient was referred to our clinic three days after a traffic accident. In radiographic examination, the right maxillary central incisor was fractured horizontally in apical thirds. Initially, following local infiltrative anesthetics, the coronal fragment was repositioned and this was radiographically confirmed. Then the stabilization splint was applied and remained for three months. After three weeks, according to the results of the vitality tests, the right and left central incisors were nonvital. For the right central incisor, both the coronal and apical fragments were involved in the endodontic preparation. *Results*. For the right central tooth, both the coronal and apical root fragments were endodontically treated and obturated at a single visit with white mineral trioxide aggregate whilst the fragments were stabilized internally by insertion of a size 40 Hedstrom stainless-steel endodontic file into the canal. *Conclusion*. Four-year follow-up examination revealed satisfactory clinical and radiographic findings with hard tissue repair of the fracture line.

## 1. Introduction

Root fractures, defined as fractures involving dentine, cementum, and pulpal and supportive tissues (e.g., the periodontal ligament and alveolar bone), constitute only 0.5–7% of all dental injuries. Moreover, the age group between 10 and 20 years old is most likely to be affected. Horizontal root fractures are commonly observed in the maxillary anterior region and 75% of these fractures occur in the maxillary central incisors [1]. The root fractures are often clinically presented as a slightly extruded tooth; usually they are lingually displaced. The tooth is generally mobile, but the degree of mobility is frequently determined by the fracture location [2]. The fracture can be at the cervical, middle, or apical region of the root. The treatment can challenge with modalities depending on the level of the fracture line and the amount of the remaining root [3].

In most of the cases, the root canal treatment of the coronal fragment is sufficient, as the pulp in the apical fragment remains vital. On the other hand, in case of total necrosis, the root canal treatment of both fragments, the root canal treatment of the coronal fragment, and the surgical removal of the apical fragment and extraction of the coronal fragment and the root canal treatment and orthodontic extrusion of the apical fragment are the other treatment options [4]. Several factors such as degree of dislocation, stage of root formation, location of the fracture, time period between trauma and treatment, and type of trauma (displacement of the coronal fragment compared with no displacement of the coronal fragment) may affect the treatment success of horizontally fractured teeth [5].

In the teeth with a horizontal fracture, healing occurs with one of these types: healing with hard tissue, interposition of connective tissue, interposition of bone and connective tissue, and interposition of granulation tissue. As “healing with hard tissue” is the best result that is expected, interposition of the granulation tissue represents an inflammatory state, and it is unfavorable. Another two types are also considered favorable [6]. In the teeth with a horizontal fracture, the treatment principle is to prevent the movement of the coronal part and to protect the vitality of the pulp. For this purpose, splint application is recommended. At the present time, splinting with the orthodontic wire and composite resin for 1–3 months is preferred in the teeth with a root fracture.

## 2. Case Report

A 14-year-old female patient was referred to our clinic three days after a traffic accident. In intraoral diagnosis, the left maxillary lateral incisor was avulsed; the left and right maxillary central incisors were mobile in both horizontal and vertical directions.

**Figure 1:**
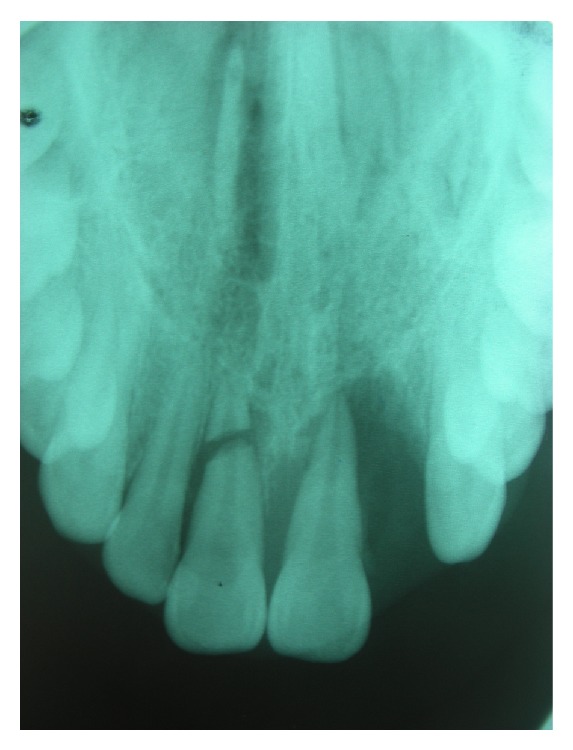
Preoperative radiograph.

**Figure 2:**
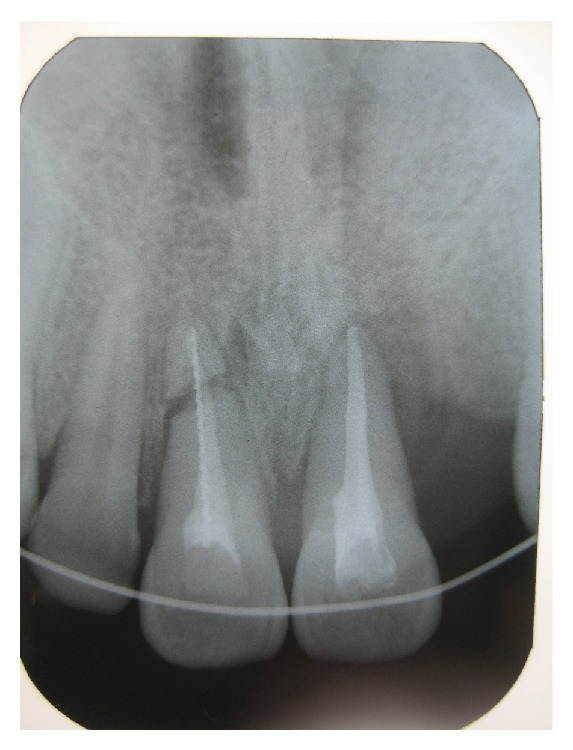
After obturation of both teeth.

**Figure 3:**
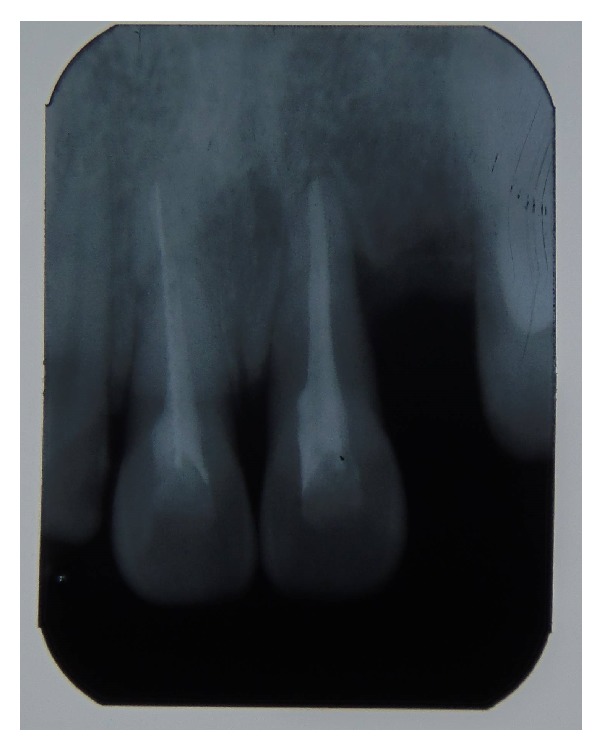
A periapical radiograph after four years follow-up.

In radiographic examination, the right maxillary central incisor was fractured horizontally in apical thirds. The other teeth had widening periodontal space because of the extrusive luxation (Figure 1). Initially, following local infiltrative anesthetics, the coronal fragment was repositioned and this was confirmed radiographically. Then the stabilization splint was applied and remained for three months. After three weeks, according to the results of the vitality tests, the right and left central incisors were nonvital. Because of this, endodontic access of the right and left central incisors was achieved and necrotic pulp tissue was removed. For the right central incisor, both the coronal and apical fragments were involved in the endodontic preparation. The root canals were prepared to size 40 using standardized instrumentation technique. Irrigation was performed copiously with 2.5% sodium hypochlorite (NaOCl). A standardized root canal treatment using the lateral condensation technique with Gutta Percha (Diadent, USA) and root canal sealer (AH Plus, Dentsply, Konstanz, Germany) was applied to the left central incisor tooth. For the right central tooth, both the coronal and apical root fragments were endodontically treated and obturated at a single visit with white MTA (MTA-Angelus, Angelus, Brazil) whilst the fragments were stabilized internally by insertion of a size 40 Hedstrom stainless-steel endodontic file into the canal (Figure 2). After the stabilization splint was removed, the patient was recalled every 6 months during the four-year follow-up (Figure 3).

Four-year follow-up examination revealed satisfactory clinical and radiographic findings with hard tissue repair of the fracture line. The teeth were clinically free of symptom and presented physiological mobility. The radiographic examination of the root-fractured tooth showed periodontal space of a normal width, normal lamina dura connection, and hard tissue healing of the fracture line, possibly with the cementoid material. The implant treatment was planned in tooth space of the avulsed tooth as the patient reached the age of 18.

## 3. Discussion

This case report presents the root canal treatment of a tooth with a horizontal root fracture. In such cases, after proper clinical management with repositioning and splinting, the patient should be followed up periodically without a root canal treatment [1]. As the incidence of necrosis in cases of horizontal root fracture is slightly over 20%, it has been suggested that an immediate endodontic intervention should be avoided provided that there are no clinical and/or pathological signs. Furthermore, making clinical and radiographic follow-up as a choice of treatment is recommended [7, 8]. In approximately 25% of adult patients with a horizontal root fracture, permanent pulpal necrosis occurs in the coronal fragment and requires a root canal treatment [6, 9]. When pulp necrosis develops, the apical part of the fractured tooth usually remains vital. The apical pulpal circulation is often not disrupted, as the apical fragment is not displaced [10].

A root-fractured tooth requires an adequate initial intervention and periodic evaluations [1]. Realizing the healing patterns of the root fracture is imperative for a successful treatment. Diastasis between fragments has a great effect on both healing the fracture line and the pulpal necrosis [11]. Properly treated teeth with horizontal root fractures have a good prognosis.

In this case, an endodontic instrument was used to fix the separated root fragments. For the same reason, other clinicians have used a metal pin or a dental post, which was positioned passively inside the root canal together with endodontic cement [12]. In a study, it has been stated that healing was more frequent in horizontal root-fractured teeth when endodontic treatment of only the coronal fragment is accomplished compared to when endodontic treatment of both of the fragments is accomplished [6].

In this case, MTA was used for root canal filling of horizontal root-fractured teeth. In the previous studies a higher fracture resistance, higher clinical and radiographic success, absence of signs of clinical and radiographic failure, greater amount of hard tissue formation, and a lower level of inflammation have been observed when MTA-filled root canals are compared with root canals filled with other materials [13, 14]. Hence, MTA is commonly preferred in the root canal treatment of the horizontal root fracture, because its use can enhance the outcome of the treatment. This case report demonstrates a good long-term outcome when MTA was used in a horizontal root-fractured tooth with intraradicular splinting.
